# Unique Properties of the Rabbit Prion Protein Oligomer

**DOI:** 10.1371/journal.pone.0160874

**Published:** 2016-08-16

**Authors:** Ziyao Yu, Pei Huang, Yuanhui Yu, Zhen Zheng, Zicheng Huang, Chenyun Guo, Donghai Lin

**Affiliations:** The Key Laboratory for Chemical Biology of Fujian Province, MOE Key Laboratory of Spectrochemical Analysis & Instrumentation, College of Chemistry and Chemical Engineering, Xiamen University, Xiamen 361005, China; INRA Centre de Jouy-en-Josas, FRANCE

## Abstract

Prion diseases, also known as transmissible spongiform encephalopathies (TSEs), are a group of fatal neurodegenerative disorders infecting both humans and animals. Recent works have demonstrated that the soluble prion protein oligomer (PrP^O^), the intermediate of the conformational transformation from the host-derived cellular form (PrP^C^) to the disease-associated Scrapie form (PrP^Sc^), exerts the major neurotoxicity *in vitro* and *in vivo*. Rabbits show strong resistance to TSEs, the underlying mechanism is unclear to date. It is expected that the relative TSEs-resistance of rabbits is closely associated with the unique properties of rabbit prion protein oligomer which remain to be addressed in detail. In the present work, we prepared rabbit prion protein oligomer (recRaPrP^O^) and human prion protein oligomer (recHuPrP^O^) under varied conditions, analyzed the effects of pH, NaCl concentration and incubation temperature on the oligomerization, and compared the properties of recRaPrP^O^ and recHuPrP^O^. We found that several factors facilitated the formation of prion protein oligomers, including low pH, high NaCl concentration, high incubation temperature and low conformational stability of monomeric prion protein. RecRaPrP^O^ was formed more slowly than recHuPrP^O^ at physiological-like conditions (< 57°C, < 150 mM NaCl). Furthermore, recRaPrP^O^ possessed higher susceptibility to proteinase K and lower cytotoxicity *in vitro* than recHuPrP^O^. These unique properties of recRaPrP^O^ might substantially contribute to the TSEs-resistance of rabbits. Our work sheds light on the oligomerization of prion proteins and is of benefit to mechanistic understanding of TSEs-resistance of rabbits.

## Introduction

The transmissible spongiform encephalopathies (TSEs), also known as prion diseases, are the ongoing threat to humans and animals, which result from the accumulation of the misfolded form of the normal cellular prion protein (PrP^C^) [1]. Prion diseases are the fatal neurodegenerative diseases, including variant Creutzfeldt-Jakob disease (vCJD) in human, bovine spongiform encephalopathy (BSE) in cattle, scrapie in sheep and goat [1–4]. So far the underlying pathogenic mechanisms of prion diseases are still unclear. The conformational transformation of the prion protein is believed to be the critical event in prion pathogenesis. Previous works have demonstrated that the conformation of the prion protein could be converted from the cellular PrP^C^ state into the non-infectious amyloid state under acidic and neutral pH conditions in the presence of detergents or denaturants [5–10]. Note that the non-infectious PrP amyloid state is distinctly different from the infectious PrP^Sc^ state, although both states show the properties of PK-resistance. According to the “protein only” hypothesis, the conformational transformation from the α-helix-rich form PrP^C^ into the β-sheet-rich form PrP^Sc^ plays a crucial role in the pathogenesis of prion diseases [11]. PrP^Sc^ was originally defined by Prusiner as an insoluble proteinase K-resistant form of PrP detected in prion-infected tissue, and could aggregate into amyloid rods [12]. As a template for the conformational transformation, PrP^Sc^ had previously been considered to be the pathogenic factor of prion diseases for many years [13]. Recent studies demonstrated that the insoluble fibrillar form PrP^Sc^ did not exhibit significant neurotoxicity *in vitro*, while the soluble β-sheet-rich prion oligomer as an intermediate of the conformational transformation, exerted significant neurotoxicity *in vitro* and *in vivo* [14, 15], and exhibited neurotoxicity stronger than the fibrillar counterpart *in vivo* [15]. These results suggest that oligomeric PrP^O^ is one of the pathogenic factors for the TSEs.

Rabbits are one of the few mammalian animals reported to be relatively resistant to TSE agents, which could survive with oral inoculation of the human kuru and CJD agents or scrapie agents isolated from sheep and mice [16]. Although human and rabbit prion proteins share very high sequence identity [17], recent investigations showed that the specific domains beyond PrP-H2H3 of rabbit prion protein remarkably affected its misfolding [18, 19]. Previous works suggested that multiple amino acid residues throughout the rabbit PrP^C^ sequence significantly contribute to the inability of the cellular form being converted to the scrapie isoform and thereby are closely associated with TSEs-resistance of rabbits [17, 20–22]. Considering the conformational transformation is mostly dependent on the structural stability of the host prion protein, it could be expected that distinct TSEs-susceptibility difference between human and rabbit is closely associated with their abilities of conformational conversion.

The prion protein oligomer is the critical factor in the pathogenesis of prion diseases. Several works have been previously performed to access the oligomerization of PrP^C^ from TSEs-susceptible species including mouse, human, sheep and hamster [7, 15, 23, 24]. These works demonstrated that α-helix-rich PrP^C^ could be converted into β-sheet-rich PrP^O^ before forming PrP^Sc^ or amyloidogenic fibril, and the oligomeric PrP^O^ exhibited significant neurotoxicity [15, 25, 26]. To our best knowledge, few work has been reported on the oligomerization of TSEs-resistant rabbit prion protein. It is expected that the properties of rabbit prion protein oligomer might distinctly differ from those of TSEs-susceptible prion protein oligomers. Thus, the comparison of prion protein oligomerization between the TSEs-susceptible human PrP^C^ and TSEs-resistant RaPrP^C^ would provide valuable clues for mechanistic understanding of TSEs-resistance.

In the present study, we conducted the comparison of the unique properties of rabbit prion protein oligomer (recRaPrP^O^) with those of human prion protein oligomer (recHuPrP^O^). We prepared oligomeric recRaPrP^O^ and recHuPrP^O^ proteins from monomeric recRaPrP^C^_91-228_ and recHuPrP^C^_91-230_ proteins under acidic pH condition without detergents or denaturants. Moreover, we analyzed the effects of pH, NaCl, and incubation temperature on prion protein oligomerization, and compared the oligomerization rate, proteinase K-resistance and cytotoxicity between recRaPrP^O^ and recHuPrP^O^. Our results may be helpful for in-depth understanding of the oligomerization process of prion proteins, and also give hints to the molecular mechanism underlying the TSEs-resistance of rabbits.

## Materials and Methods

### Oligomeric prion protein preparation

Plasmid construction, protein expression and purification were almost the same as described previously [21, 27]. The protein concentration was determined using NanoVue plus (GE Healthcare, USA) at 280 nm. The extinction coefficient of 22×10^3^ M^-1^ cm^-1^ was calculated based on the amino acid sequences of HuPrP^C^_91-230_ and RaPrP^C^_91-228_ using the web-based tool provided by ExPasy. The purified prion proteins were diluted to 40 μM in a buffer (20 mM NaOAc, 150 mM NaCl, 0.02% NaN_3_, pH 4.0). The proteins were incubated at 47°C for 160 min. To exploit the effect of NaCl on prion oligomerization, sodium acetate buffers were used with NaCl concentrations of 50 mM, 100 mM, 150 mM and 200 mM, respectively. Data were processed with the software Unicorn 5.2. The oligomer level was calculated as the ratio of the area of the oligomer peak to the total area in the elution profile.

### Size exclusion chromatography

Size exclusion chromatography was performed using ӒKTA fast protein liquid chromatography (FPLC) equipment (GE Healthcare, USA) with a Superdex G-75 column (M_r_: 3–70 kDa). The column has been calibrated with standard proteins such as ribonuclease A (~ 13.7 kDa, ~ 14 mL). Five column volumes of elution buffer were used to equilibrate the column prior to the experiments. The flow rate was 0.3 mL/min and the protein elution was monitored by UV-absorption at 280 nm.

### Dynamic light scattering measurement

Dynamic light scatting (DLS) was performed on Malvern-dynamic light scattering Zetasizer Nano-ZS90 (Malvern Instruments, UK). Prion monomers and oligomers were prepared at 0.65 mg/mL and loaded into a 1-cm-path UV-transparent disposable cuvette. DLS data were collected at 25°C with 30 measurements for each sample.

### Circular dichroism spectroscopy

All circular dichroism (CD) spectra were recorded on a Jasco J-810 spectropolarimeter (Jasco, Japan) interfaced with a Peltier-type temperature control at 25°C. The spectrum was an average of three consecutive scans and blanked with respective buffers. Far-UV CD spectra were collected in the wavelength range of 200–260 nm using 1-mm path length on samples containing 0.2 mg/mL protein in a buffer (20 mM NaOAc, pH 5.5). The spectra were recorded in continuous scanning mode at a scanning rate of 50 nm/min with a band width of 1 nm. Samples for the equilibrium unfolding were diluted to 10 μM in the buffer (20 mM NaOAc, pH 5.5). The range of urea concentrations up to 10 M was used with 0.5 M gradient. The temperature range was from 25 to 95°C. Two apparent thermodynamic parameters, C_m_ and T_m_, were used to describe the conformational stability of the proteins. Here, C_m_ was the concentration of urea required to denature 50% of the proteins, and T_m_ represented the observed midpoint of the thermal transition.

### Proteinase K assays

Oligomeric recRaPrP^O^ and recHuPrP^O^ proteins (40 mM) were incubated with proteinase K (Darmstadt, Germany) in a buffer (10 mM Tris-HCl, 2 mM CaCl_2_, pH 7.4) for 0–80 min at 37°C. Digestion was stopped by adding 3 mM phenylmethylsulfonyl fluoride (PMSF). The products of digestion were analyzed by 15% SDS-PAGE.

### Cell culture

Human glioblastoma cell line U87 was purchased from American Type Culture Collection (ATCC, USA). Cells were grown in Dulbecco’s modified Eagle’s medium (DMEM) (Sigma) containing 1% penicillin G and streptomycin (Sigma, USA) and 10% fetal bovine serum (FBS, USA). Cells were cultured in a humidified incubator with 5% CO_2_ at 37°C.

### Cytotoxicity evaluation

U87 Cells were treated in the absence or presence of prion protein oligomers for 48 h. The cytotoxicity was measured quantitatively by the (3-(4,5-dimethylthiazol-2-yl)-5-(3-carboxymethoxyphenyl)-2-(4-sulfophenyl)-2H tetra-zolium (MTS) (Promega, USA) assay with CellTiter 96®AQ_ueous_ One Solution Cell Proliferation Assay Kit (Promega, USA). The absorbance value at 490 nm was proportional to the number of living cells. U87 cells were seeded at a density of 1×10^4^ cells/well in a 96-well poly-Dlysine–coated plate. After 24 h of culture, cells were incubated with prion protein oligomers for 48 h. For controls, the cells were either left untreated or exposed to an equivalent volume of PBS and vehicle solution.

## Results

### Preparation of prion protein oligomers

**Fig 1:**
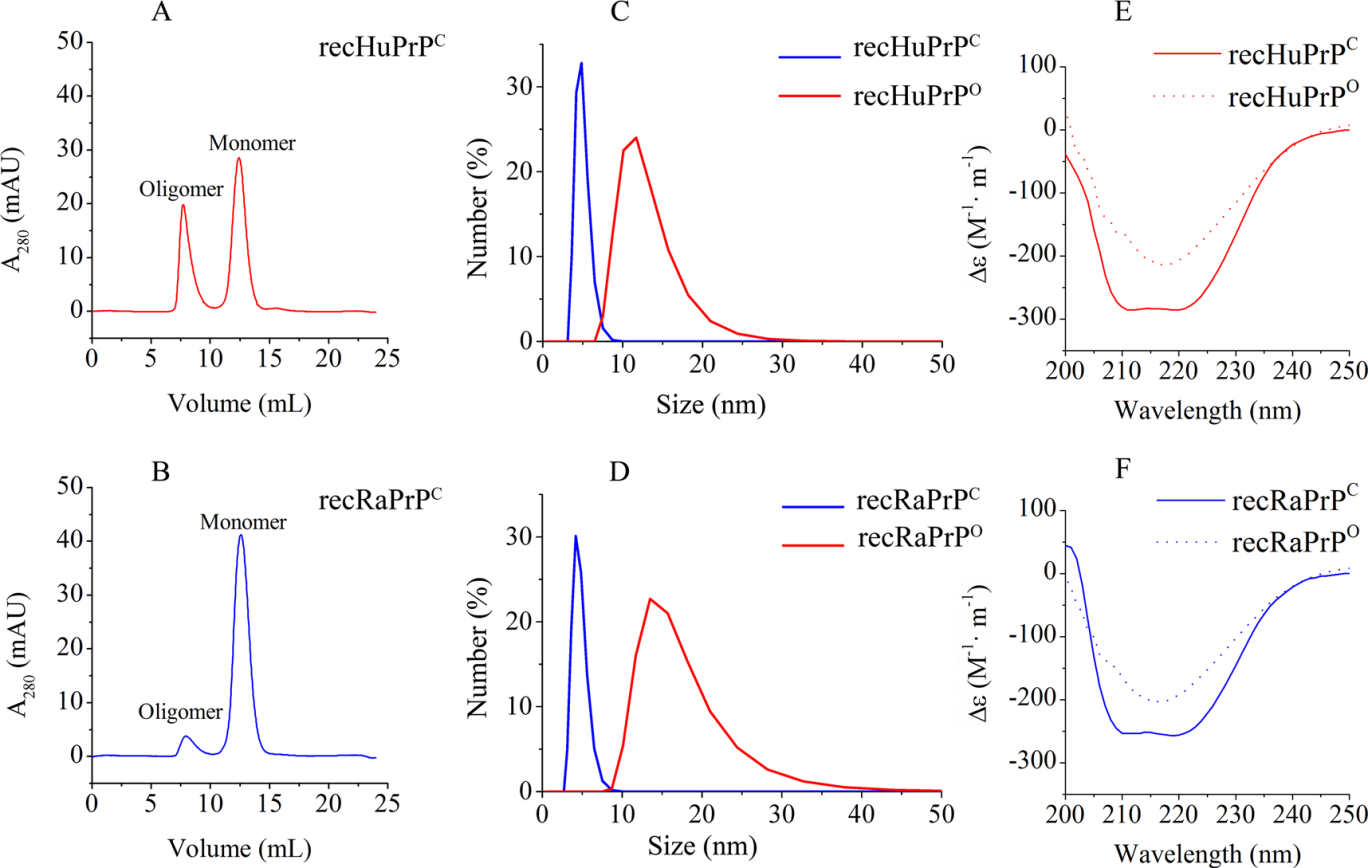
Characterization of monomer and oligomer properties for human and rabbit prion proteins. Oligomerization of human prion protein (A) and rabbit prion protein (B) in the buffer (20 mM NaOAc, 150 mM NaCl, pH 4.0) was monitored by gel filtration chromatography. The prion protein oligomers were prepared with a buffer (20 mM NaOAc, 150 mM NaCl, pH 4.0) at 47°C. Particle sizes of both human prion protein (C) and rabbit prion protein (D) were analyzed by DLS spectroscopy. Secondary structures of human prion protein (E) and rabbit prion protein (F) were detected by Far-UV CD spectroscopy. Both DLS and CD experiments were performed at 25°C. The buffer used for prion monomers contained 20 mM NaOAc, pH 5.5.

To prepare oligomeric recPrP^O^ proteins, we incubated monomeric PrP^C^ proteins in the buffer (20 mM NaOAc, pH 4.0) without or with 150 mM NaCl at 47°C for 20 min, and monitored the oligomeric statuses of the prion proteins using gel filtration chromatography. Only one single peak at 15 mL (corresponding to the elution volume of monomeric recPrP^C^) was detected in the buffer without NaCl, while two peaks at 7 and 13 mL were observed in the presence of 150 mM NaCl (Fig 1A and 1B). The peak at 13 mL was associated with monomeric recPrP^C^ protein with an apparent molecular weight of ~ 16 kDa. The peak corresponding to oligomeric recPrP^O^ was eluted in the void volume (7 mL), implying that the apparent molecular weight was higher than the fractionation limit of the Superdex G-75 column (70 kDa for globular proteins).

With the DLS spectroscopy, the diameters of recHuPrP^O^, recHuPrP^C^, recRaPrP^O^ and recRaPrP^C^ were evaluated to be 12.4, 4.8, 16.3 and 4.6 nm, respectively (**Fig 1C and 1D**). These results confirmed that the particle sizes of prion oligomers were in the range of 6–30 nm [10, 14, 23]. Moreover, the DLS results indicated that the particle size of recRaPrP^O^ was larger than that of recHuPrP^O^ **(S1 Fig)**, whereas the particle size of monomeric recRaPrP^C^ was almost the same as that of recHuPrP^C^.

In addition, we also analyzed the secondary structures of the prion protein monomers and oligomers by Far-UV CD spectroscopy. Two negative peaks were observed at 208 nm and 222 nm for monomeric recHuPrP^C^ and recRaPrP^C^ (**Fig 1E and 1F**), indicating that the prion protein monomers adopted α-helix-rich structures. The Far-UV CD spectra were consistent with those reported by previous works [21, 22, 28]. However, only one negative peak was detected at 217 nm for oligomeric recHuPrP^O^ and recRaPrP^O^ (**Fig 1E and 1F**), suggesting that the prion protein oligomers adopted β-sheet-rich structures.

### NaCl concentration affects prion oligomerization rate

**Fig 2:**
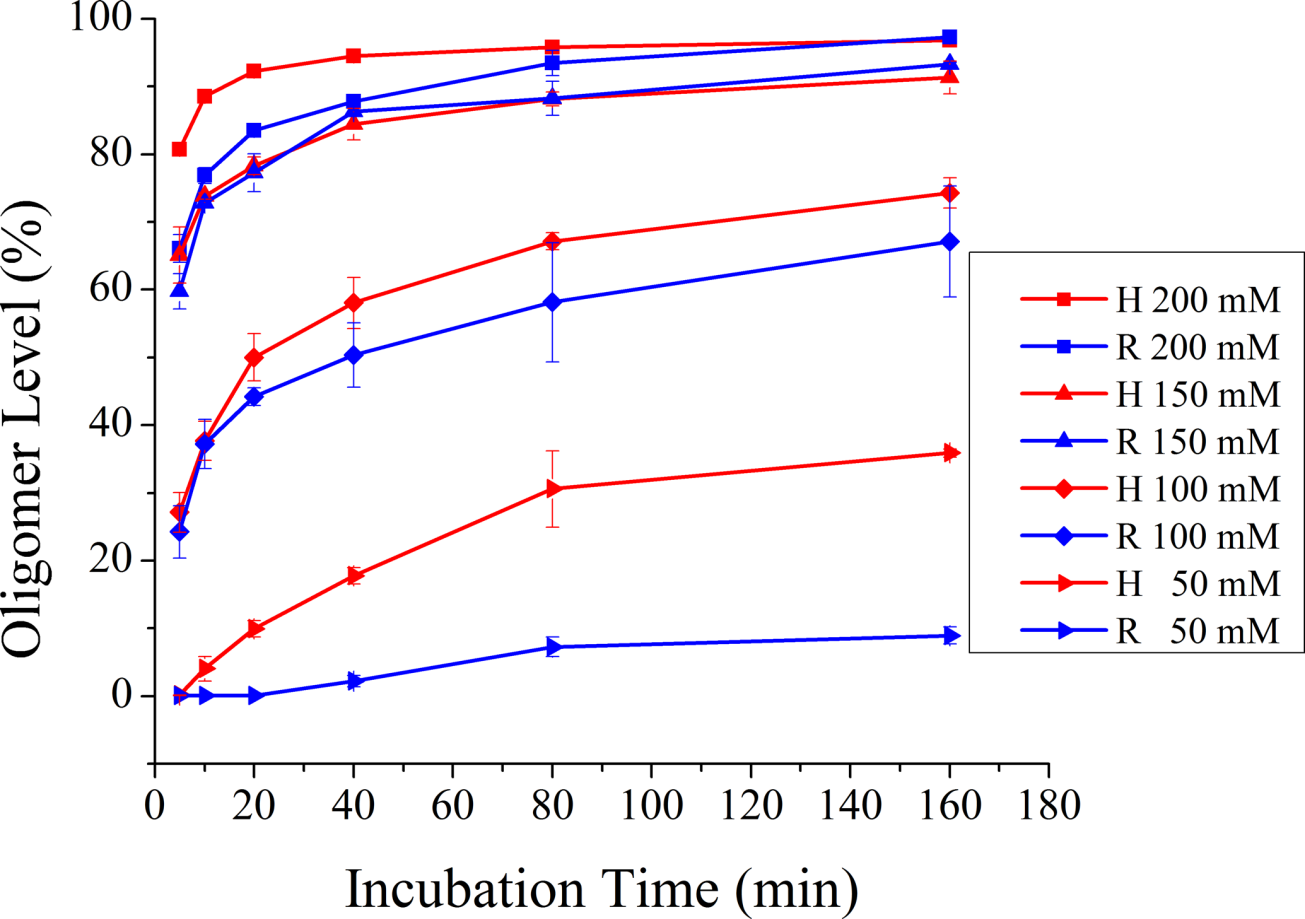
NaCl concentration-dependent oligomerization of recHuPrP^C^ and recRaPrP^C^ monitored by gel filtration chromatography. The oligomerization experiments of prion proteins were conducted in the buffer (20 mM NaOAc, 50–200 mM NaCl, pH 4.0) at 57°C (n = 3; Error bars, S.D.).

The NaCl concentration is closely correlated with the formation of prion protein oligomers [9]. Here we analyzed the effect of NaCl concentration on prion protein oligomerization by incubating monomeric recHuPrP^C^ and recRaPrP^C^ proteins at NaCl concentrations ranging from 50 mM to 200 mM for 5–160 min. We observed that the oligomerization of recHuPrP^C^ and recRaPrP^C^ was NaCl concentration-dependent (**Fig 2, S2 Fig)**. High NaCl concentrations (150 mM, 200 mM) led to larger oligomization rates and higher oligomer levels than low NaCl concentrations (50 mM, 100 mM). When incubated at 50 mM NaCl (pH 4.0, 57°C) for 160 min, about 35.9% of recHuPrP^C^ and 8.9% of recRaPrP^C^ monomers were converted into recHuPrP^O^ and recRaPrP^O^, respectively. However, when incubated at high NaCl concentrations (150 mM, 200 mM) for only 40 min, more than 80% of prion protein monomers were converted into the oligomers (**Fig 2, S1 Table**). After incubation at 200 mM NaCl concentration for 160 min, the oligomer levels of recHuPrP^O^ and recRaPrP^O^ reached up to 96.8% and 97.3%, respectively, implying that high NaCl concentrations could significantly promote the formation of prion protein oligomers.

Furthermore, we observed distinctly different NaCl concentration-dependences on both oligomerization rates and oligomer levels between recHuPrP^C^ and recRaPrP^C^ (**Fig 2**). RaPrP^C^ showed smaller oligomerization rates and lower oligomer levels than recHuPrP^C^ when incubated at low NaCl concentrations (50 mM, 100 mM). However, both monomeric proteins exhibited similar oligomerization rates and almost identical oligomer levels when incubated at high NaCl concentrations (150 mM, 200 mM). The oligomerization curves show that the saturation level of recHuPrP^O^ is significantly higher than that of recRaPrP^O^ at low NaCl concentration, but both saturation levels are almost identical at high NaCl concentration. These results indicated that NaCl concentration significantly influenced both the oligomerization rate and oligomer level of prion proteins. It could be an efficient approach to prepare prion protein oligomers at high NaCl concentration.

### NaCl destabilizes human and rabbit PrP^C^

Previous works have been performed to address the role of NaCl in the conformational conversion and aggregation of prion proteins [9, 28, 29]. Apetri et al. have reported that salt could significantly reduce the thermodynamic stability of recHuPrP^C^ in urea-induced denaturation experiments [29]. Our results showed that recRaPrP^C^ oligomerized much more slowly than recHuPrP^C^ at lower NaCl concentration (50 mM, 100 mM). However, at higher NaCl concentration (150 mM, 200 mM), the oligomerization rate of recRaPrP^C^ was nearly the same as that of recHuPrP^C^ (**Fig 2**). Therefore, it could be speculated that the conformational stabilities of recHuPrP^C^ and recRaPrP^C^ are different, on which NaCl concentrations have distinct effects.

**Fig 3:**
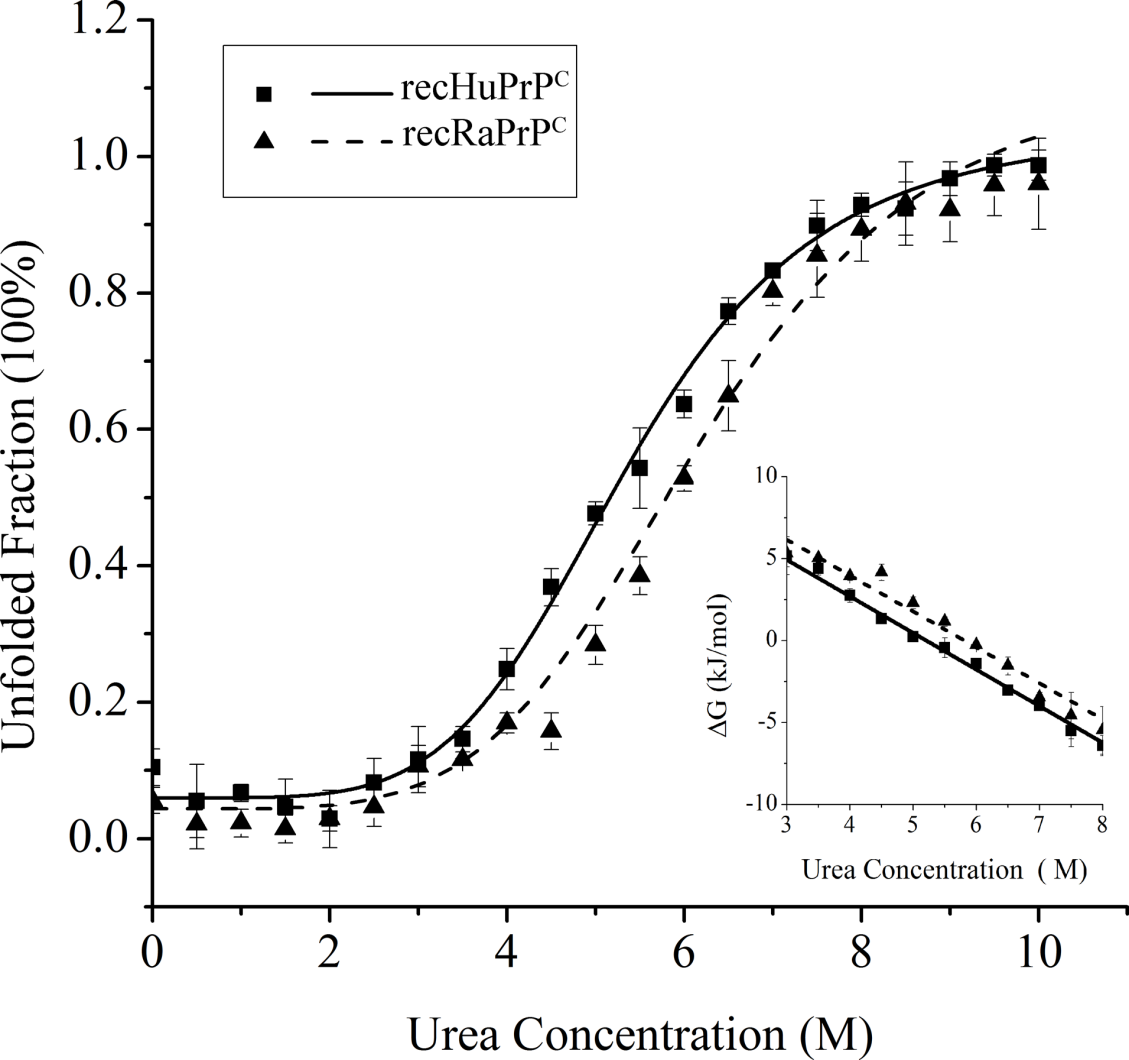
Urea-induced unfolding transitions of recRaPrP^C^ and recHuPrP^C^ proteins analyzed at 25°C by Far-UV CD spectroscopy. The buffer contained 20 mM NaOAc, pH 5.5. The unfolded fraction calculated from Δε at 222 nm is plotted as a function of the urea concentration. Insert: ΔG (kJ/mol) versus the urea concentration.

**Fig 4:**
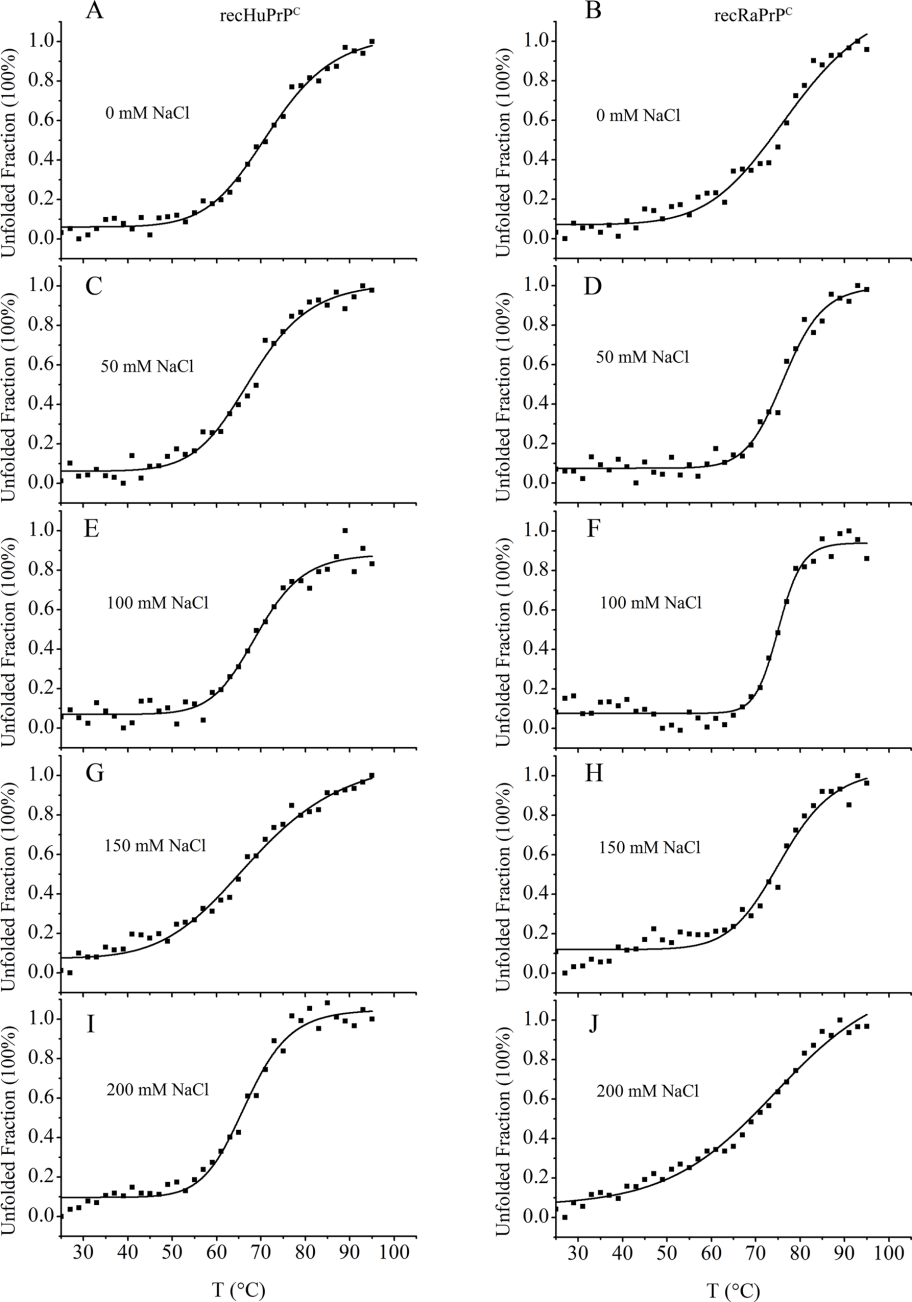
Thermal-induced unfolding transitions of recRaPrP^C^ and recHuPrP^C^ proteins analyzed by Far-UV CD spectroscopy. The buffer contained 20 mM NaOAc, 0–200 mM NaCl, pH 5.5. The unfolded fraction calculated from Δε at 222 nm is plotted as a function of temperature.

To compare the conformational stabilities of recRaPrP^C^ and recHuPrP^C^, we analyzed the urea-induced and thermal-induced unfolding transitions of both prion protein monomers using Far-UV CD spectroscopy, and determined apparent thermodynamic parameters for the equilibrium unfolding (**Figs 3 and 4, Tables 1 and 2**). The midpoint denaturant concentration C_m_ was determined to be 5.38±0.06 M for HuPrP^C^ and 6.24±0.24 M for recRaPrP^C^, respectively (**Table 1**; 20 mM NaOAc, 0 mM NaCl, pH 5.5, 25°C). The midpoint denaturation temperature T_m_ was measured to be 71.59±0.56°C for recHuPrP^C^ and 76.46±1.45°C for recRaPrP^C^, respectively (**Table 2**; 0 mM NaCl, pH 5.5). These results indicated that recHuPrP^C^ possessed a conformational stability lower than recRaPrP^C^.

**Table 1:** Apparent thermodynamic parameters associated with urea-induced unfolding transitions of recHuPrP^C^ and recRaPrP^C^ at 25°C. The buffer contained 20 mM NaOAc, pH 5.5. ΔG^H2O^_N→U_ is designated as the apparent free energy of unfolding extrapolated to zero concentration of denaturant, m_N→U_ is the cooperativity of the unfolding transition, and C_m_ is the concentration of urea required to denature 50% of the protein. The CD spectrum was an average of three consecutive scans. Each experiment was repeated in triplicate for each sample.

	ΔG^H2O^_N→U_ (kJ/mol)	m _N→U_ (kJ/mol/M)	C_m_ (M) (mean ± SD)	*p*-value
**recHuPrP**^**C**^	11.74 ± 0.37	-2.22 ± 0.06	5.38 ± 0.06	0.0038
**recRaPrP**^**C**^	13.26 ± 0.41	-2.27 ± 0.07	6.24 ± 0.24	

**Table 2:** Apparent thermodynamic parameters associated with thermal-induced unfolding transitions of recHuPrP^C^ and recRaPrP^C^. The buffer contained 20 mM NaOAc, 0–200 mM NaCl, pH 5.5. ΔG^0°C^_N→U_ is designated as the apparent free energy of unfolding extrapolated to 0°C, m_N→U_ is the cooperativity of the unfolding transition, and T_m_ is the temperature at the midpoint of unfolding. The CD spectrum was an average of three consecutive scans. One experiment was conducted for each sample.

	ΔG^0°C^_N→U_(kJ/mol)		m _N→U_ (kJ/mol/M)		T_m_ (°C) (mean ± SD)		*p*-value
NaCl (mM)	recHuPrP^C^	recRaPrP^C^	recHuPrP^C^	recRaPrP^C^	recHuPrP^C^	recRaPrP^C^	
**0**	18.61±0.89	22.36±1.60	-0.24±0.01	-0.32±0.02	71.59±0.56	76.46±1.45	0.056
**50**	17.51±1.07	21.78±2.37	-0.27±0.02	-0.29±0.03	69.07±0.68	76.38±0.51	0.001
**100**	15.57±1.40	18.94±2.09	-0.24±0.02	-0.25±0.03	67.72±0.59	75.18±0.38	<0.0001
**150**	14.37±0.93	17.54±2.91	-0.22±0.02	-0.24±0.05	66.81±0.94	75.62±1.00	0.0004
**200**	12.97±0.94	14.91±0.89	-0.19±0.02	-0.23±0.02	66.62±0.49	74.39±2.00	0.0028

Furthermore, we compared the effects of NaCl concentrations on conformational stabilities of recHuPrP^C^ and recRaPrP^C^. The conformational stabilities were decreased with increasing NaCl concentrations. When 50 mM NaCl was added, the T_m_ value of recHuPrP^C^ was significantly decreased from 71.59±0.56°C to 69.07±0.68°C, while that of recRaPrP^C^ was 76.38±0.51°C without observable change. As the NaCl concentration was increased to 200 mM, the T_m_ value of recHuPrP^C^ was significantly decreased to 66.62±0.49°C, whereas that of recRaPrP^C^ was 74.39±2.00°C with insignificant change. These data indicated that NaCl affected the conformational stability of recHuPrP^C^ significantly than that of recRaPrP^C^, suggesting that recRaPrP^C^ was more stable than recHuPrP^C^.

### Both acidic pH condition and high temperature significantly promoted prion protein oligomerization

Extensive experimental reports have demonstrated that prion proteins tended to aggregate at acidic pH [8, 30, 31]. When recHuPrP^C^ and recRaPrP^C^ were incubated in either sodium acetate buffer (pH 5.5, 150 mM NaCl) or sodium phosphate buffer (pH 7.4, 150 mM NaCl) at 67°C for 160 min, almost no HuPrP^O^ and RaPrP^O^ oligomers were formed (data not shown). However, when incubated in sodium acetate buffer (pH 4.0, 150 mM NaCl) at 67°C for 40 min, almost all the recHuPrP^C^ and recRaPrP^C^ monomers were converted into the oligomers (**S2 Table**). These results indicated that low pH environment facilitated the oligomerization of prion proteins.

**Fig 5:**
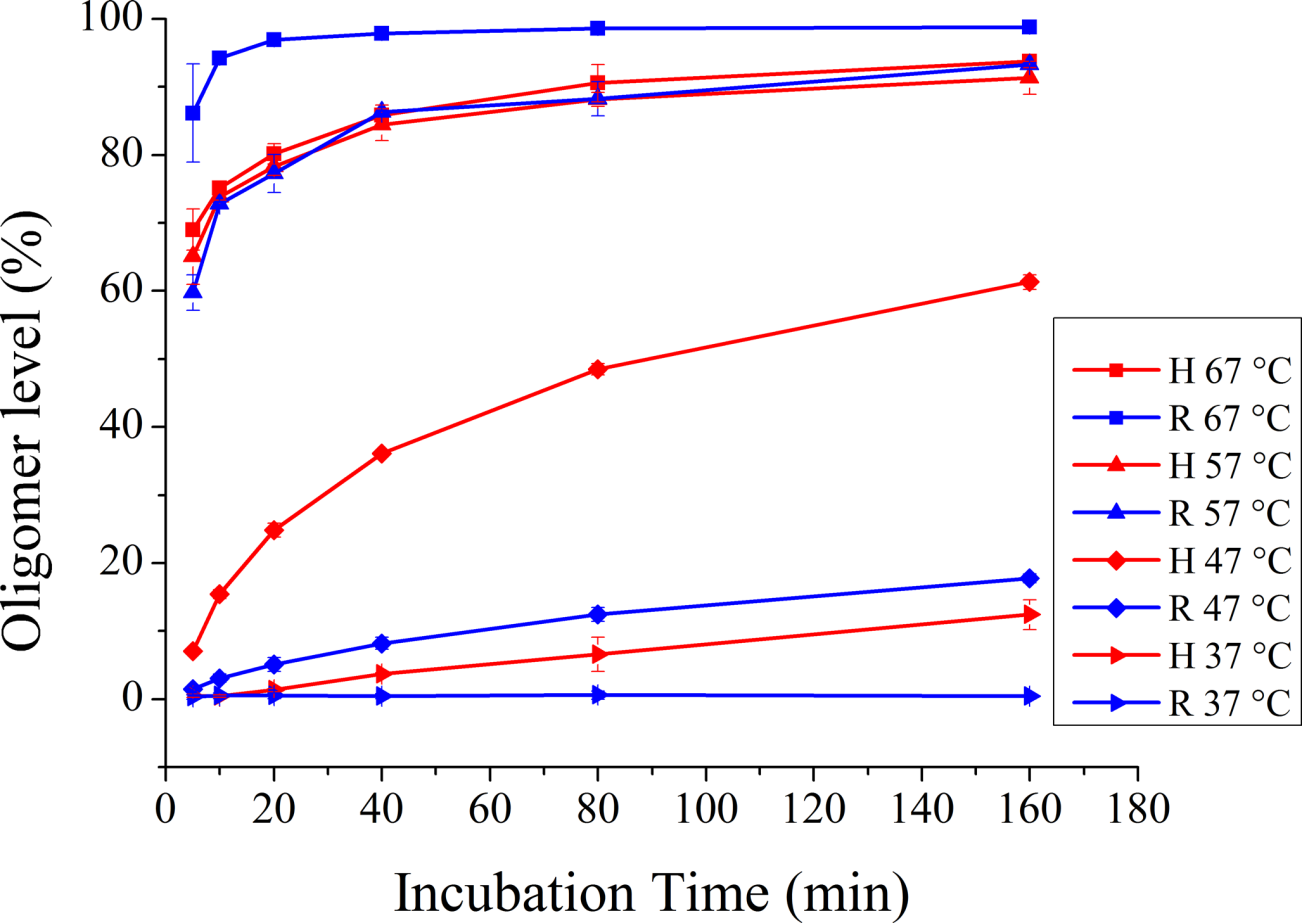
Incubation temperature-dependent oligomerization of recHuPrP^C^ and recRaPrP^C^ monitored by gel filtration chromatography. The oligomerization experiments of prion proteins were conducted in a buffer (20 mM NaOAc, 150 mM NaCl, pH 4.0) at 37–67°C (n = 3; Error bars, S.D.).

On the other hand, previous works have accessed thermal-induced oligomerization of prion proteins [16, 23, 32]. In the present work, we explored the effect of incubation temperature on prion protein oligomerzation by incubating recHuPrP^C^ and recRaPrP^C^ proteins in sodium acetate (pH 4.0, 150 mM NaCl) at temperatures ranging from 37°C to 67°C for 5–160 min. We found the oligomerization of both prion proteins was temperature-dependent and time-dependence (**S2 and S3 Figs**). High incubation temperatures (57°C, 67°C) led to larger oligomization rates and higher oligomer levels than low incubation temperatures (37°C, 47°C). When incubated at 37°C (pH 4.0, 150 mM NaCl) for 160 min, about 12.4% of recHuPrP^C^ and 0.4% of recRaPrP^C^ monomers were converted into recHuPrP^O^ and recRaPrP^O^ oligomers (**Fig 5, S2 Table**). However, after incubation at 67°C (pH 4.0, 150 mM NaCl) for 160 min, 93.8% of recHuPrP^C^ and 98.8% of recRaPrP^C^ monomers were converted into oligomers (**Fig 5, S2 Table**). These results implied that high incubation temperature could significantly promote the formation of prion protein oligomers.

In addition, significant temperature-dependent differences in both oligomerization rates and oligomer levels were observed between recHuPrP^C^ and recRaPrP^C^. Compared with recHuPrP^C^, recRaPrP^C^ showed much smaller oligomerization rates and much lower oligomer levels when incubated at low temperatures (37°C, 47°C; **Fig 3**). However, both prion proteins exhibited similar oligomerization rates and almost identical oligomer level when incubated at 57°C (**Fig 3**). Intriguingly, when incubated at 67°C, recRaPrP^C^ showed oligomerization rate and oligomer levels larger than recHuPrP^C^. Similarly to NaCl concentration, incubation temperature also significantly affected both the oligomerization rate and oligomer level of prion proteins.

### Proteinase K (PK) -resistance and cytotoxicity of recHuPrP^O^ and recRaPrP^O^

**Fig 6:**
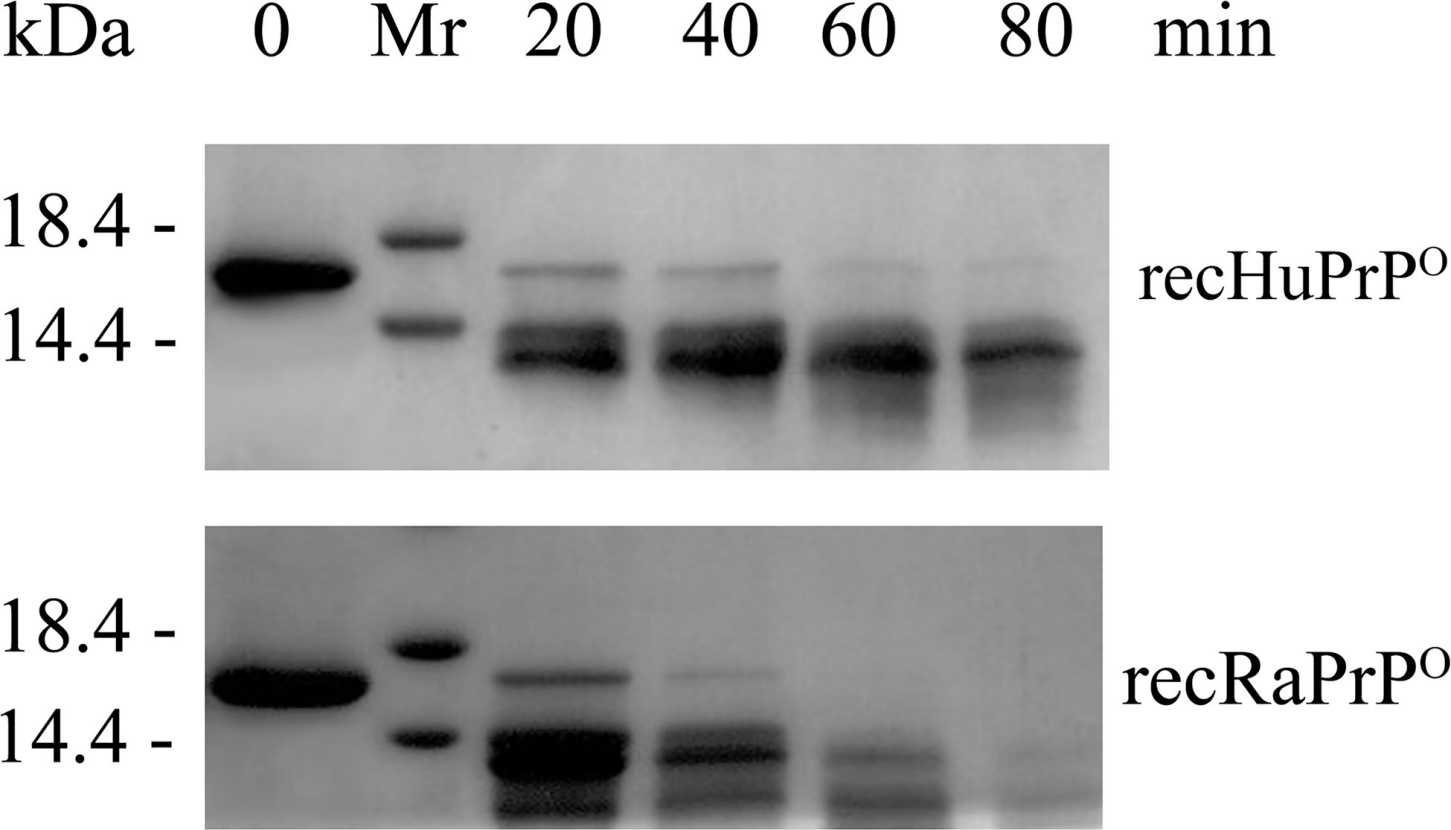
15% SDS-PAGE analysis of protease K digestion of recHuPrP^O^ and recRaPrP^O^ proteins. The oligomeric proteins (40 mM) were digested by protease K (2 μg/ml) for 0–80 min, in a buffer (10 mM Tris-HCl, 2 mM CaCl_2_, pH 7.4) at 37°C.

The previous work has demonstrated that ovine and murine prion protein oligomers possessed the characteristics of PrP to PrP^Sc^ conversion intermediates such as partial protease resistance, and showed neurotoxicity *in vitro* on primary cultures of neurons and *in vivo* after subcortical stereotaxic injection [15]. Herein, we compare the PK-resistance and cytotoxicity of recHuPrP^O^ and recRaPrP^O^. The concentrations of PK from 0.2 to 20 μg/ml were previously used to degrade PrP aggregations [18, 30]. As an intermediate of PrP^C^→PrP^Sc^ conversion, PrP^O^ could be easily degraded by PK. Therefore, we chose a relative low concentration (2 μg/ml) of PK to conduct the PK digestion experiments. The PK digestion of recHuPrP^O^ and recRaPrP^O^ is shown in Fig 6, which displays the PK-resistance difference between both prion protein oligomers. Almost all recHuPrP^O^ proteins (40 mM) were degraded by 2 μg/mL PK at 37°C in 60 min, and some proteins were still visible in 80 min (**Fig 6A**). However, nearly all recRaPrP^O^ proteins (40 mM) were degraded in 40 min, and almost no proteins were visible in 80 min (**Fig 6B**). These results suggested that recHuPrP^O^ had stronger tolerance to cellular hydrolases than recRaPrP^O^.

**Fig 7:**
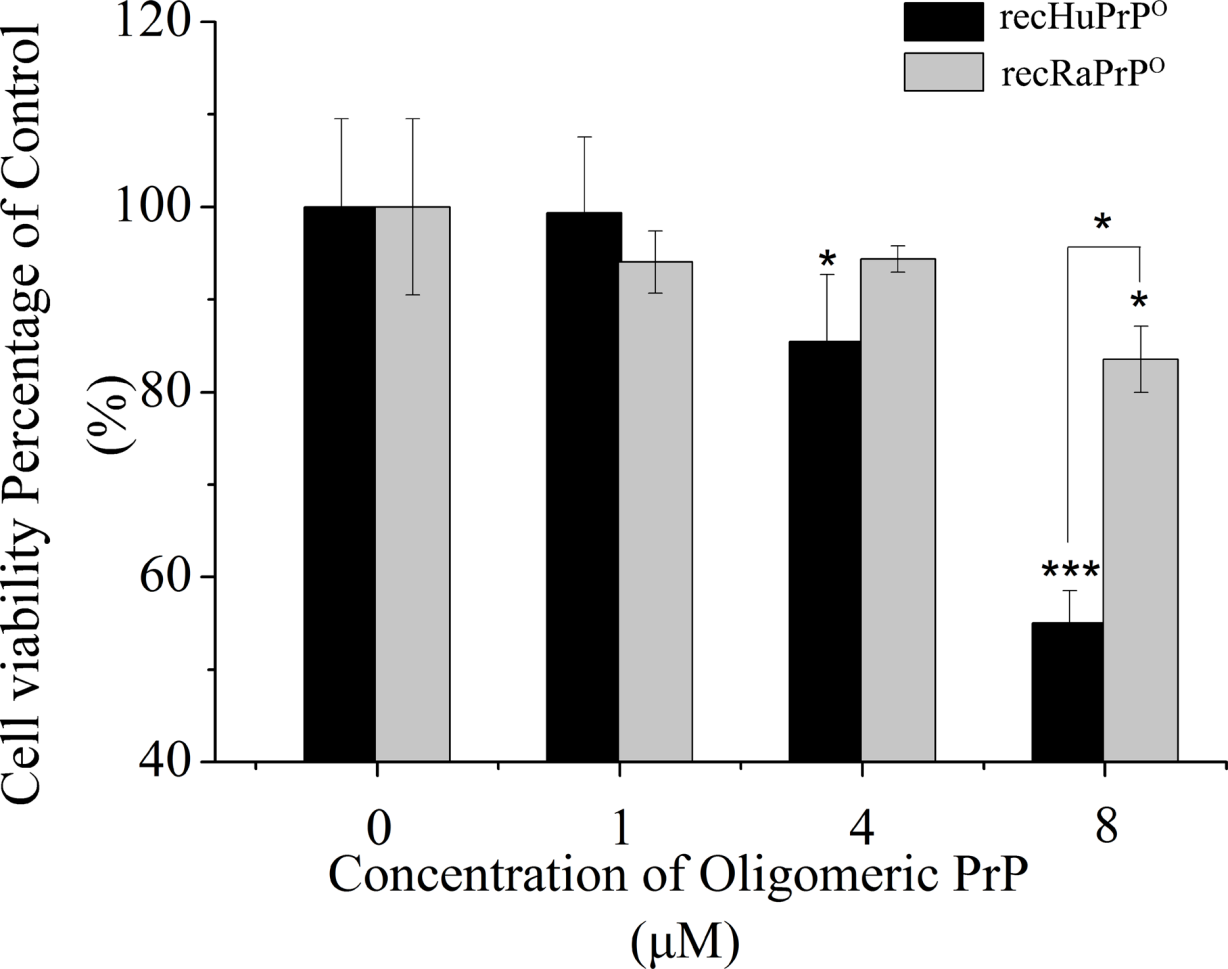
Comparison of recHuPrP^O^-induced and recRaPrP^O^-induced toxicities on human glioblastoma cell lines U87. Cells were incubated with oligomeric PrP^O^ proteins at different concentrations for 48 h (37°C). Cytotoxicity was quanitified as a function of cell viability by the MTS assay (n = 3, mean±SD; *, *p*<0.01; ***, *p*<0.001; by Multiple Comparison Test).

We evaluated the cytotoxicity of recHuPrP^O^ and recRaPrP^O^ against the gliobalstoma cell lines U87 by incubating the cells with prion protein oligomers at various concentrations (1 μM, 4 μM, 8 μM) for 48 h. We thereafter analyzed cell viabilities against oligomeric prion proteins by using MTS assay (**Fig 7**). The cells treated with 1 μM prion protein oligomers did not show distinct cytotoxicity compared with the vehicle-treated control cells after 48 h incubation. However, the cells exhibited significant cytotoxicity when the concentration of the prion protein oligomer was increased to 8 μM, at which the viability of the cells treated with recHuPrP^O^ was lower than that with recRaPrP^O^. These results indicated that the oligomeric prion proteins induced significant cytotoxicity at 8 μM, and the recHuPrP^O^-induced cytotoxicity was distinctly higher than RaPrP^O^-induced cytotoxicity.

## Discussion

As an intermediate of the conformational transformation from PrP^C^ into PrP^Sc^, soluble oligomers exerts neurotoxicity *in vitro* and *in vivo*, and are responsible for the neuron dysfunction and death in prion diseases [14, 15]. Even though several works have been previously performed to explore the oligomerization of PrP^C^ proteins from TSEs-susceptible species [32–35], the oligomerization of PrP^C^ proteins from TSEs-resistant species has not yet been exploited. The formation of soluble prion protein oligomers could reflect in some extent the potential of the occurrence of prion infection. Although rabbits are one of TSEs-resistant species, the monomeric RaPrP^C^ could be converted into PrP^Sc^ or the fibril form under specific conditions as previously reported [18, 36, 37]. It seems that the pathogenesis of prion diseases is mostly associated with the potentials of conformational transformation and PrP^C^ aggregation. In the present work, we used an efficient approach to prepare prion protein oligomers from recombinantly expressed recHuPrP^C^ (91–230) and recRaPrP^C^ (91–228) monomers, and compared their biophysical and biochemical properties with several biophysical techniques.

Our results showed that under acidic pH condition in the presence of 150 mM NaCl, the conformations of both recHuPrP^C^ and recRaPrP^C^ proteins could be converted from α-helix-rich monomers into β-sheet-rich oligomers (**Fig 1E and 1F**). Either the low pH or high temperature was not sufficient to induce the oligomerization of PrP^C^, and additional destabilizing factors (such as high NaCl concentration) were required to promote the conformational conversion of PrP^C^. Marillas et al. exhibited that the unfolding transition of recHuPrP_90-231_ at acidic pH was associated with strong salt dependence [9]. Baskakov et al. showed that the presence of NaCl significantly promoted the formation of the β-isoform of rPrP106 and mouse PrP [5, 28]. Our work demonstrated that the oligomerization of prion protein monomers was significantly NaCl concentration-dependent, incubation temperature-dependent.

In addition, our work showed that the particle size of recRaPrP^O^ was larger than that of recHuPrP^O^ (**Fig 1C and 1D**), implying that monomeric recRaPrP^C^ tended to form larger oligomers compared with monomeric recHuPrP^C^. The gel filtration experiment could be employed to determine the molecular weight which was smaller than the fractionation limit of a calibrated column. The peak corresponding to oligomeric recPrP^O^ was eluted in the void volume, implying that the apparent molecular weight was higher than the fractionation limit of the column (70 kDa for globular proteins). In such a case, the gel filtration experiments could not give the accurate molecular weights of recRaPrP^O^ and recHuPrP^O^. However, the molecular weights of both prion protein oligomers could be evaluated by DLS experiments. Previously, Cheon et al. suggested that both the total hydrophobic area and hydrogen bonds could affect the particle size of oligomeric Aβ peptide [38]. The Aβ peptide was responsible for the pathology of Alzheimer’s disease [38] as one of the neurodegenerative diseases [39]. Thus, it could be speculated that different hydrophobic areas and hydrogen bonds were potentially associated with different particle sizes of the oligomers formed by recHuPrP^C^ and recRaPrP^C^. Furthermore, in our previous work, we observed that the recRaPrP^O^ solution (100 μM) grew into white flocculent precipitation in two weeks at 4°C, while the recHuPrP^O^ solution (100 μM) remained clean even after three months (data not shown). Recent works on protein conformational diseases suggested that the fibrous amyloid of the protein, acting as a protective sink to neutralize the toxic oligomers, might be the end point of protein aggregation [40, 41]. The large prion protein oligomers might facilitate precipitation and potentially decrease their cytotoxicities.

### Conformational stability of monomeric prion proteins affects prion oligomerization rate

Expectedly, the conformational stability of prion protein monomers is closely correlated with the prion protein oligomerization rate. It is well known that hydrogen bond, van der Waals force, hydrophobic interaction, and salt bridge are the crucial factors determining the conformational stability of proteins. Our previous works showed that the numbers of hydrogen bonds and salt bridges contained in the wild-type recRaPrP^C^_91-228_ were larger than those contained in its S173N and I214V variants, indicating that the wild-type protein possessed high conformational stability [21, 22]. Moreover, NMR relaxation measurements demonstrated that both variants possessed backbone dynamics distinctly different from that of the wild-type recRaPrP^C^_91-228_, and indicated that the S173N and I214V substitutions resulted in increased backbone flexibility and decreased conformational stability in the variant proteins [21, 22]. These results suggest that the unique structural characteristics of rabbit prion protein, such as intrinsically high conformational stability and unique distribution of surface electrostatic potentials, might inhibit the conformational conversion from PrP^C^ to PrP^Sc^.

Our data showed that increasing NaCl concentration decreased the conformational stabilities of monomeric recRaPrP^C^ and recHuPrP^C^ (**Table 2**). The higher NaCl concentration was associated with the lower midpoint denaturation temperature (T_m_) of the prion protein monomers. Furthermore, the thermal-induced unfolding experiments also showed that the T_m_ value of recRaPrP^C^ was higher than that of recHuPrP^C^ at the same NaCl concentration (**Table 2**). Our results indicated that the conformational stability of recHuPrP^C^ was lower than that of recRaPrP^C^ in the absence and presence of NaCl. It could be expected that lower conformational stability potentially facilitates the formation of prion protein oligomers. This expectation was true for the case using lower NaCl concentration (50, 100 mM) rather than higher NaCl concentration (150, 200 mM). The detailed reason should be addressed further.

### Incubation temperature affects prion protein oligomerization rate

Reazei et al. analyzed the oligomerization pathway of the full-length recombinant monomeric ovine PrP (OPrP^C^), and found that OPrP^C^ irreversibly formed two well-identified soluble oligomers with high β-sheet content through heat-induction [23]. It could be speculated that the prion protein with lower conformational stability might possess larger propensity to misfold and oligomerize. Our results showed that recRaPrP^C^ possessed high conformational stability and oligomerized much more slowly than recHuPrP^C^ at physiological temperature. Contrarily, when incubated at 67°C recRaPrP^C^ oligomerized much rapidly than recHuPrP^C^. Our work supports the MD simulation results reported by Zhang’s work, which suggests that rabbit prion does not have higher conformational stability than human prion protein at higher temperature, although rabbit prion protein is more stable than human prion protein at lower temperature [42]. These results reveal the complexity of thermal effects on prion proteins.

### Potential relative TSEs-resistance mechanism of rabbits

Regarding to the pathological mechanism of neurodegenerative diseases such as TSEs, it was recently demonstrated that the cytotoxicity of aggregated proteins was mostly resulted from the pre-fibrillar forms, oligomers [15, 25, 43, 44]. The protein aggregates could impair the cellular functions due to the resistance to enzymolysis [45, 46]. They could directly interplay with cellular components and saturate the cellular clearance pathway [15, 47]. Therefore, prion protein oligomers are believed to be important pathogenic factors in prion diseases [48]. Although the protein misfolding cyclic amplification (PMCA) technique could be applied to overcome the species barrier in rabbits [37], rabbits are still thought to be one of the relatively TSEs-resistant species [49, 50]. In the present work, recRaPrP^O^ showed cytotoxicity lower than recHuPrP^O^, potentially due to its higher susceptibility to cellular proteinases. As a matter of fact, our PK-resistance experiments described above have demonstrated that recRaPrP^O^ proteins were degraded by proteinase K more readily than recHuPrP^O^, implying that recRaPrP^O^ has weaker PK-resistance than recHuPrP^O^. These results suggest that recRaPrP^O^ could be easily cleaned up by cellular proteinases before accumulating to the toxicity level. Further experiments should be conducted to support this viewpoint.

Notably, the “species barrier” effect could make contribution to the difference between the cytotoxicity of recRaPrP^O^ and that of recHuPrP^O^, which were evaluated with the human glioblastoma cell line. As showed in previous studies, the “species barrier” effect is an important factor contributing to the species’ resistance to the TSEs. Expectedly, recRaPrP^O^ might not show full toxicity to the human glioblastoma cell line due to the “species barrier” effect. Unfortunately, we have not yet obtained the required rabbit cell line from ATCC or other cell banks for evaluating the cytotoxicity of recRaPrP^O^.

It is true that until now no evidence has been provided to show that the cytotoxicity of oligomeric PrP^O^ involves in prion propagation. Nevertheless, it could be expected that the toxicity of prion protein oligomers would affect the pathological process of prion disease. Our results showed that recRaPrP^O^ was less toxic than recHuPrP^O^. Less cytotoxicity might mean less damage to cell functions or less influence to the pathological process. Expectedly, the property that recRaPrP^O^ had less cytotoxicity potentially made contribution to TSEs- resistance of rabbits.

Summarily, the present work demonstrates that recRaPrP^C^ proteins oligomerize more slowly than recHuPrP^C^ proteins in physiological-like environments. Moreover, the weaker PK-resistance of recRaPrP^O^ implies that shorter time is required to clean up recRaPrP^O^ compared with recHuPrP^O^. This characteristic of recRaPrP^O^ potentially prevents it from reaching enough toxicity level to impair normal cellular functions. Our results suggest that the relative TSEs-resistance of rabbits is closely associated with the unique properties of both monomeric recRaPrP^C^ (higher conformational stability, smaller oligomerzation rate, lower oligomer level), and oligomeric recRaPrP^O^ (weaker PK-resistance, lower cytotoxicity). Our work is helpful for understanding the relative TSEs-resistance of rabbits, and sheds light on the molecular mechanisms of prion diseases.

## Supporting Information

S1 Fig Comparison of the sizes of recHuPrP^O^ and recRaPrP^O^ oligomers measured by DLS. DLS data were collected at 25°C with 30 measurements for each sample (n = 3, mean ± SD, *, *p*<0.05).
(TIF)

S2 Fig NaCl concentration-dependent and temperature-dependent oligomerization of recHuPrP^C^ and recRaPrP^C^ monitored by gel filtration chromatography. Oligomerization of recHuPrP^C^(A)and recRaPrP^C^(B)incubated for 20 min at 57°C in a buffer (20 mM NaOAc, pH 4.0) containing 50–200 mM NaCl. Oligomerization of recHuPrP^C^ (C) and recRaPrP^C^ (D) incubated for 20 min at 37–67°C in a buffer (20 mM NaOAc, pH 4.0) containing 150 mM NaCl.
(TIF)

S3 Fig Incubation time-dependent oligomerization of recHuPrP^C^ and recRaPrP^C^ proteins monitored by gel filtration chromatography. (A) RecHuPrP^C^ incubated at 37°C; (B) RecRaPrP^C^ incubated at 37°C; (C) RecHuPrP^C^ incubated at 47°C; (D) RecRaPrP^C^ incubated at 47°C. The buffer contained 20 mM NaOAc, 150 mM NaCl, pH 4.0.
(TIF)

S1 Table Mean oligomer levels of human and rabbit prion proteins incubated at 57°C. The buffer contained 20 mM NaOAc, 50–200 mM NaCl, pH 4.0.
(DOC)

S2 Table Mean oligomer levels of human and rabbit prion proteins incubated at 37–67°C. The buffer contained 20 mM NaOAc, 150 mM NaCl, pH 4.0.
(DOC)

## Abbreviations

CD: circular dichroism
DLS: dynamic light scattering analyses
recHuPrP^C^: recombinantly expressed human PrP^C^
recHuPrP^O^: human recPrP^O^
PK: proteinase K
PMCA: protein misfolding cyclic amplification
recRaPrP^C^: recombinantly expressed rabbit PrP^C^
recRaPrP^O^: rabbit recPrP^O^
PrP: prion protein
PrP^C^: cellular form of prion protein
PrP^O^: prion protein oligomer
recPrP^O^: oligomer of recombinantly expressed prion protein
PrP^Sc^: scrapie form of prion protein
TSEs: transmissible spongiform encephalopathies
