# Supplementary figures and images for: Unique Properties of the Rabbit Prion Protein Oligomer
Source: PLoS One. 2016 Aug 16;11(8):e0160874. doi: 10.1371/journal.pone.0160874 (PMC4987043; doi:10.1371/journal.pone.0160874)

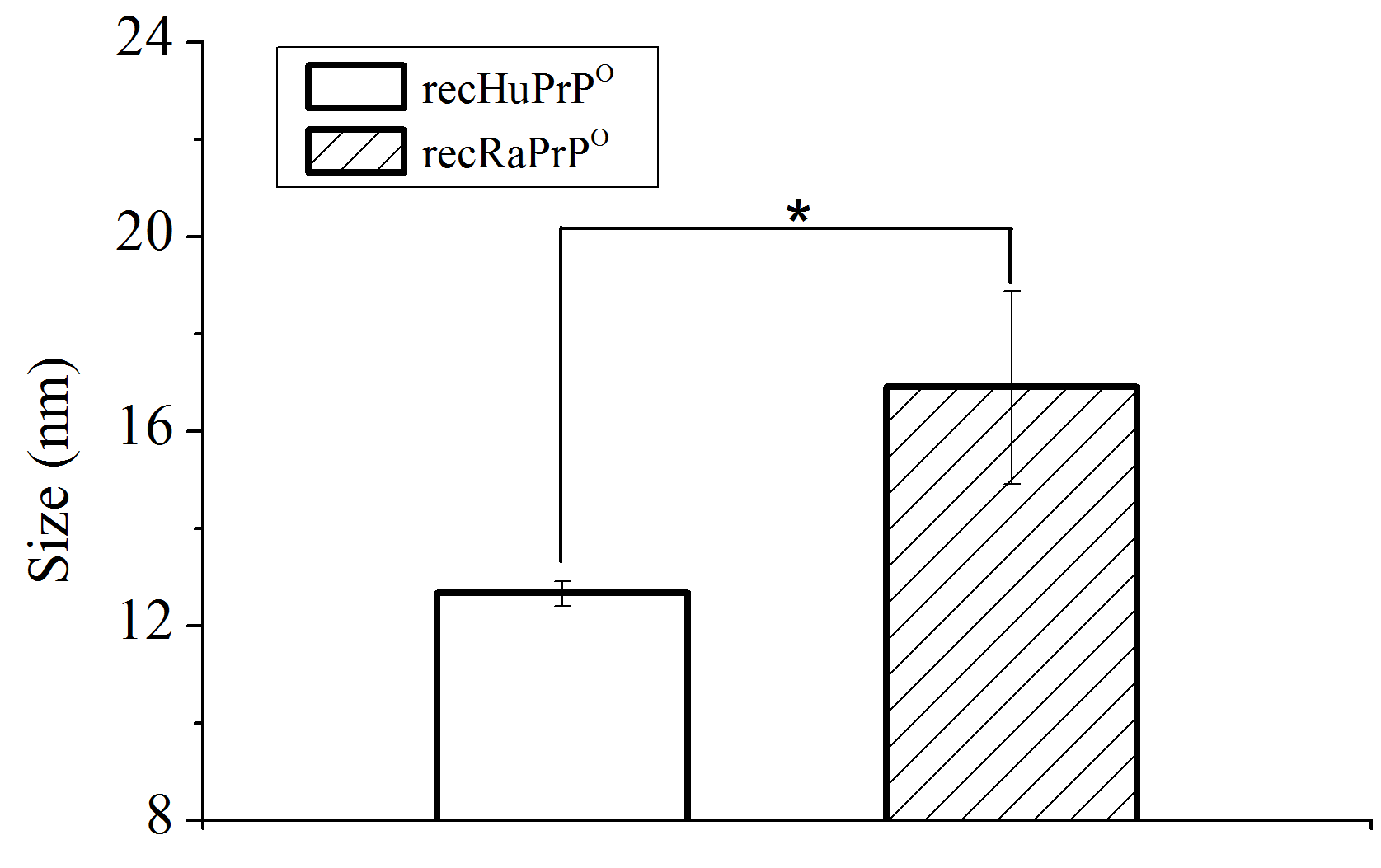

Supplement: S1 Fig — DLS data were collected at 25°C with 30 measurements for each sample (n = 3, mean ± SD, *, p<0.05). (TIF) [file pone.0160874.s001.tif]

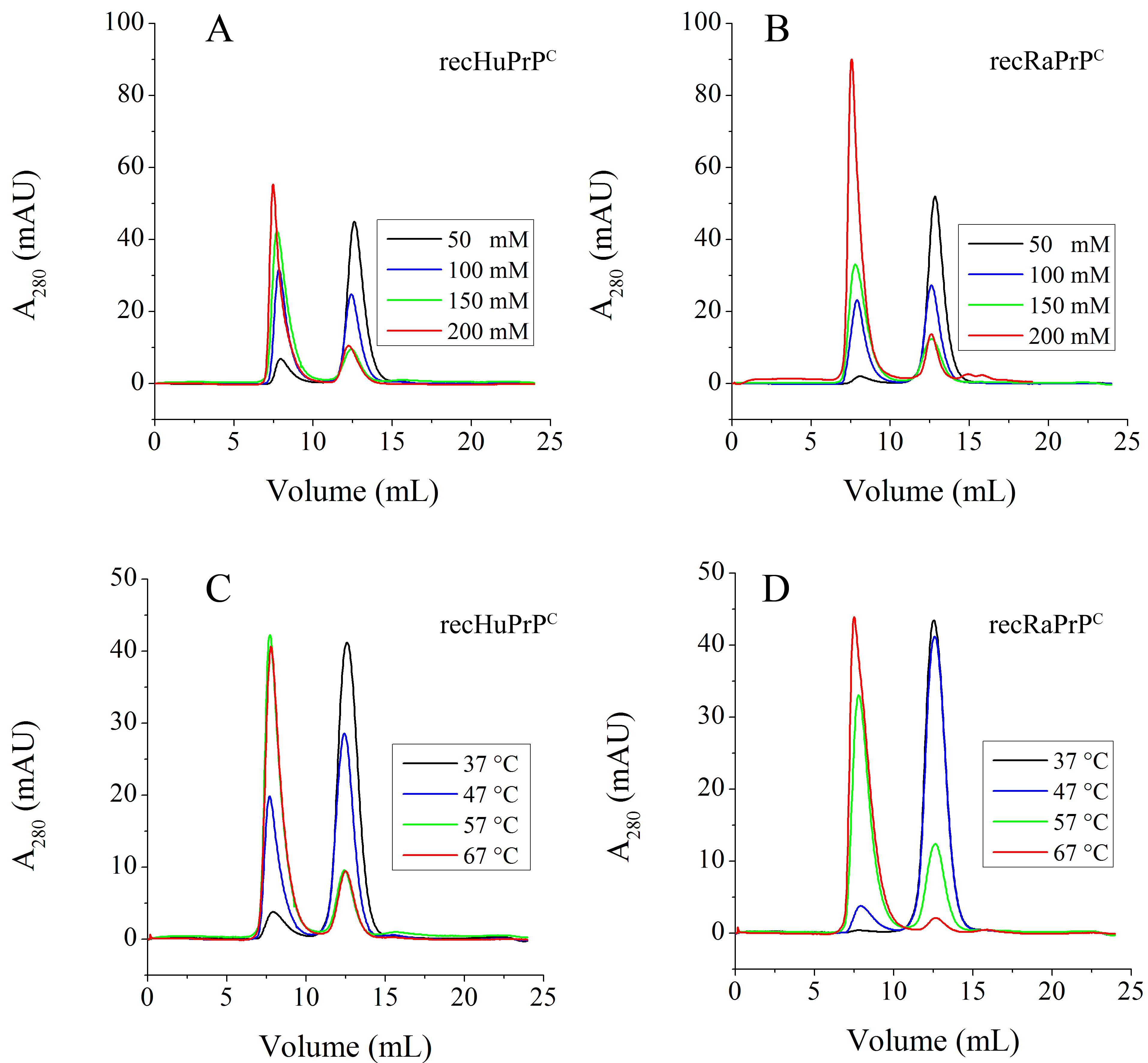

Supplement: S2 Fig — Oligomerization of recHuPrPC(A)and recRaPrPC(B)incubated for 20 min at 57°C in a buffer (20 mM NaOAc, pH 4.0) containing 50–200 mM NaCl. Oligomerization of recHuPrPC (C) and recRaPrPC (D) incubated for 20 min at 37–67°C in a buffer (20 mM NaOAc, pH 4.0) containing 150 mM NaCl. (TIF) [file pone.0160874.s002.tif]

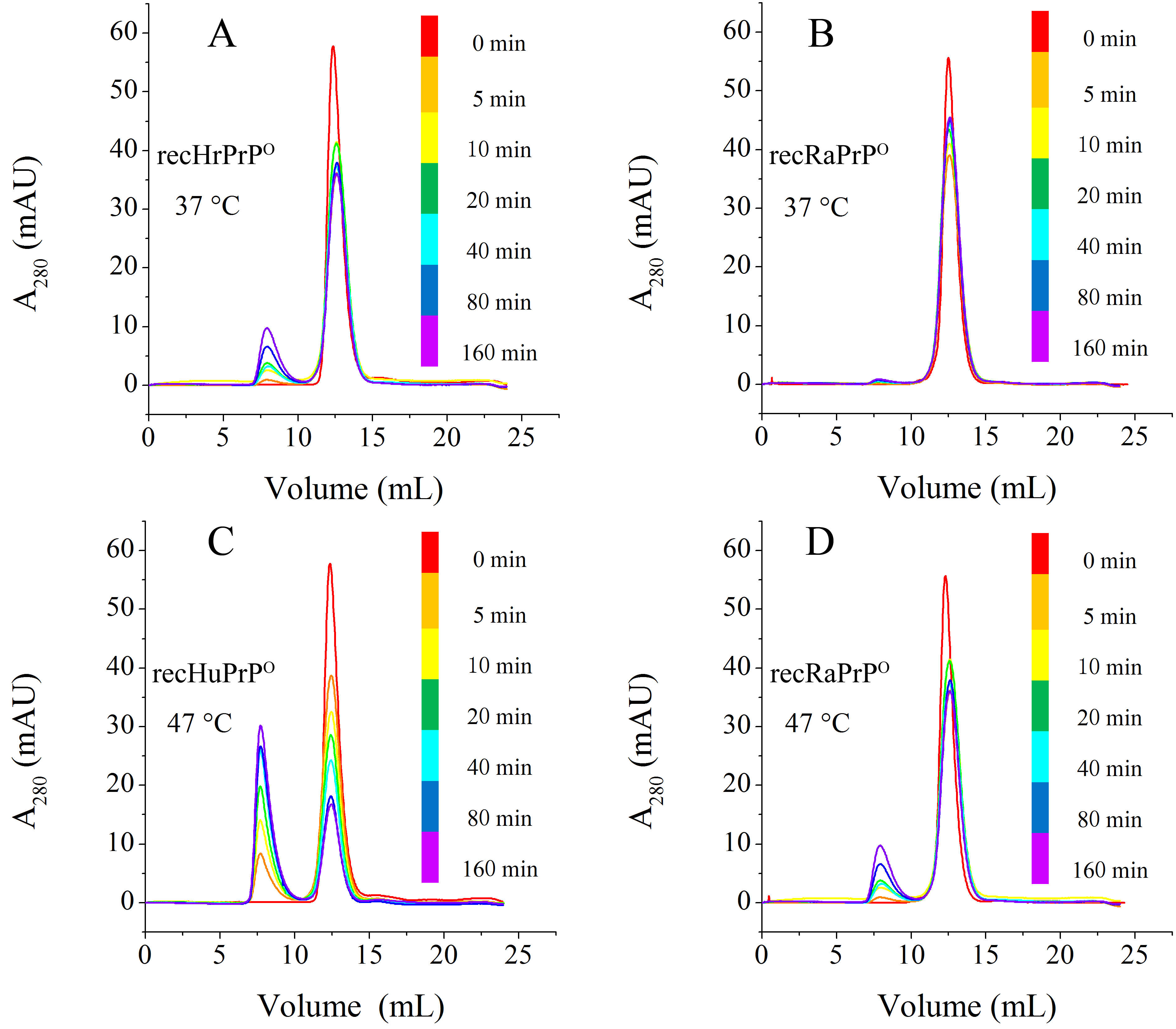

Supplement: S3 Fig — (A) RecHuPrPC incubated at 37°C; (B) RecRaPrPC incubated at 37°C; (C) RecHuPrPC incubated at 47°C; (D) RecRaPrPC incubated at 47°C. The buffer contained 20 mM NaOAc, 150 mM NaCl, pH 4.0. (TIF) [file pone.0160874.s003.tif]
