# Supplementary material for: Unique Properties of the Rabbit Prion Protein Oligomer
Source: PLoS One. 2016 Aug 16;11(8):e0160874. doi: 10.1371/journal.pone.0160874 (PMC4987043; doi:10.1371/journal.pone.0160874)
Supplement: S1 Table — The buffer contained 20 mM NaOAc, 50–200 mM NaCl, pH 4.0. (DOC) [file pone.0160874.s004.doc]

**Table S1. Mean oligomer levels of human and rabbit prion proteins incubated at 57 °C.** The buffer contained 20 mM NaOAc, 50-200 mM NaCl, pH 4.0.

|  | **50 mM** | | **100 mM** | | **150 mM** | | **200 mM** | |
| --- | --- | --- | --- | --- | --- | --- | --- | --- |
|  | 40 min | 160 min | 40 min | 160 min | 40 min | 160 min | 40 min | 160 min |
| **recHuPrP^O^** | 17.7% | 35.9% | 58.1% | 74.3% | 84.5% | 91.3% | 94.5% | 96.8% |
| **recRaPrP^O^** | 2.2% | 8.9% | 50.4% | 67.1% | 86.3% | 93.3% | 87.8% | 97.3% |
