# Supplementary material for: Unique Properties of the Rabbit Prion Protein Oligomer
Source: PLoS One. 2016 Aug 16;11(8):e0160874. doi: 10.1371/journal.pone.0160874 (PMC4987043; doi:10.1371/journal.pone.0160874)
Supplement: S2 Table — The buffer contained 20 mM NaOAc, 150 mM NaCl, pH 4.0. (DOC) [file pone.0160874.s005.doc]

**Table S2. Mean oligomer levels of human and rabbit prion proteins incubated at 37-67 °C.**

The buffer contained 20 mM NaOAc, 150 mM NaCl, pH 4.0.

|  | **37** °C | | **47** °C | | **57** °C | | **67 °C** | |
| --- | --- | --- | --- | --- | --- | --- | --- | --- |
|  | 40 min | 160 min | 40 min | 160 min | 40 min | 160 min | 40 min | 160 min |
| **recHuPrP^O^** | 3.7% | 12.4% | 36.1% | 61.3% | 84.5% | 91.3% | 85.9% | 93.8% |
| **recRaPrP^O^** | 0.5% | 0.4% | 8.2% | 17.7% | 86.3% | 93.3% | 97.9% | 98.8% |
